# Identification of the Real Hub Gene and Construction of a Novel Prognostic Signature for Pancreatic Adenocarcinoma Based on the Weighted Gene Co-expression Network Analysis and Least Absolute Shrinkage and Selection Operator Algorithms

**DOI:** 10.3389/fgene.2021.692953

**Published:** 2021-08-20

**Authors:** Qihang Yuan, Jie Ren, Zhizhou Wang, Li Ji, Dawei Deng, Dong Shang

**Affiliations:** ^1^Department of General Surgery, First Affiliated Hospital of Dalian Medical University, Dalian, China; ^2^Clinical Laboratory of Integrative Medicine, First Affiliated Hospital of Dalian Medical University, Dalian, China; ^3^Department of Oncology, First Affiliated Hospital of Dalian Medical University, Dalian, China; ^4^Department of Gastroenterology, Dongzhimen Hospital, Beijing University of Chinese Medicine, Beijing, China

**Keywords:** pancreatic adenocarcinoma, weighted gene co-expression network analysis, hub gene, least absolute shrinkage and selection operator, prognostic signature

## Abstract

**Background**: Pancreatic adenocarcinoma (PAAD) has a considerably bad prognosis, and its pathophysiologic mechanism remains unclear. Thus, we aimed to identify real hub genes to better explore the pathophysiology of PAAD and construct a prognostic panel to better predict the prognosis of PAAD using the weighted gene co-expression network analysis (WGCNA) and the least absolute shrinkage and selection operator (LASSO) algorithms.

**Methods**: WGCNA identified the modules most closely related to the PAAD stage and grade based on the Gene Expression Omnibus. The module genes significantly associated with PAAD progression and prognosis were considered as the real hub genes. Eligible genes in the most significant module were selected for construction and validation of a multigene prognostic signature based on the LASSO-Cox regression analysis in The Cancer Genome Atlas and the International Cancer Genome Consortium databases, respectively.

**Results**: The brown module identified by WGCNA was most closely associated with the clinical characteristics of PAAD. Scaffold attachment factor B (SAFB) was significantly associated with PAAD progression and prognosis, and was identified as the real hub gene of PAAD. Moreover, both transcriptional and translational levels of SAFB were significantly lower in PAAD tissues compared with normal pancreatic tissues. In addition, a novel multigene-independent prognostic signature consisting of SAFB, SP1, and SERTAD3 was identified and verified. The predictive accuracy of our signature was superior to that of previous studies, especially for predicting 3- and 5-year survival probabilities. Furthermore, a prognostic nomogram based on independent prognostic variables was developed and validated using calibration curves. The predictive ability of this nomogram was also superior to the well-established AJCC stage and histological grade. The potential mechanisms of different prognoses between the high- and low-risk subgroups were also investigated using functional enrichment analysis, GSEA, ssGSEA, immune checkpoint analysis, and mutation profile analysis.

**Conclusion**: SAFB was identified as the real hub gene of PAAD. A novel multigene-independent prognostic signature was successfully identified and validated to better predict PAAD prognosis. An accurate nomogram was also developed and verified to aid in the accurate treatment of tumors, as well as in early intervention.

## Introduction

Pancreatic adenocarcinoma (PAAD) has a high incidence rate and primarily originates from the pancreatic exocrine cells (Pothuraju et al., 2018). The 5-year survival rate for pancreatic cancer ranges from 2 to 9% due to its rapid metastasis, high degree of malignancy, and difficulty in being diagnosed early (Rawla et al., 2019). PAAD has been continuously researched in the last two decades, and many biomarkers have been verified to be useful for predicting progression and prognosis; nevertheless, patients still have a dismal prognosis (Zhou et al., 2018). Hence, novel signatures for prognosis prediction are warranted for early intervention and individualized management of PAAD. Real hub genes are also warranted to better understand the pathophysiology of PAAD.

With the widespread adoption of gene microarrays and RNA-sequencing technology, bioinformatics analyses have identified a large collection of biomarkers for better predicting the occurrence, progression, and prognosis of various cancers in recent years (Türei et al., 2015; Li et al., 2019a; Gan et al., 2020; Gu et al., 2020). For instance, the research by Li et al. (2021) identified and verified a specific glycolysis-related prognostic model for patients with PAAD (risk score=0.1755 * KIF20A −0.1400 * CHST2 +0.0214 * MET). Moreover, Xu et al. (2021) constructed and validated a novel prognostic signature for patients with PAAD based on the expression of N6-methyladenosine (m6A) regulator genes (Risk score=0.233 * KIAA1429 −0.087 * METTL3 −0.132 * METTL14 −0.035 * YTHDF1 −0.0286 * ALKBH5). However, most researchers have failed to focus on the considerable interconnection between genes when constructing prognostic signatures, and thus, the weighted gene co-expression network analysis (WGCNA) was developed. WGCNA provides a new approach in performing higher-resolution analysis, which can more accurately predict hub genes in a disease, thus, providing a novel field of vision for the exploration of disease pathophysiology and the construction of disease prognostic signatures (Panahi and Hejazi, 2021). For instance, in 2021, Farhadian et al. (2021) highlighted the pivotal role of GJA1, AP2A2, and NPAS3 in the lactation process using WGCNA algorithms. Our previous study also identified TLR7 as a candidate gene for stomach adenocarcinoma (STAD) *via* the WGCNA algorithm, and this could help predict the progression and prognosis of STAD and shed new light on its immunotherapy (Yuan et al., 2021b).

Here, we performed WGCNA and integrated high-connection genes into the same modules. All modules have been associated with the clinical phenotype, and the module most associated with the histological grade of PAAD was subsequently obtained. After a strand of screening tests, the scaffold attachment factor B (SAFB) was identified as an independent predictor of outcome and was correlated with the tumor immune microenvironment. In addition to these, the genes in the brown module were preserved for further construction of a specific multigene prognostic signature using the least absolute shrinkage and selection operator (LASSO)-Cox regression analysis. Interestingly, our results implied that the predictive accuracy of our signature was superior to the prognostic model constructed by Li et al. (2021) and Xu et al. (2021), especially for predicting 3- and 5-year survival probabilities of patients with PAAD. The potential mechanisms of survival differences between the high- and low-risk subgroups were also explored through functional enrichment analysis, gene set enrichment analysis (GSEA), single-sample gene set enrichment analysis (ssGSEA), immune checkpoint analysis, and mutation profile analysis.

## Materials and Methods

### Construction of Co-expression Modules

The PAAD mRNA expression profiles of GSE32676 used by Donahue et al. (2012) and that of GSE42952 used by Van den Broeck et al. (2012) were obtained from the Gene Expression Omnibus (GEO) database.^1^ Both GSE32676 and GSE42952 have been based on the same platform (GPL570) and contained 40 tumor samples in total. Data were preprocessed *via* the “affy” R package. The “sva” R package was further implemented to batch normalize the raw data from different datasets. Then, the top 25% most variable genes as per the analysis of variance (5,414 genes) were selected for constructing the WGCNA in R (Langfelder and Horvath, 2008). The “GoodSamplesGenes” function in the “WGCNA” package was applied to confirm the quality of the raw data. Next, we constructed an adjacency matrix through Pearson correlation analysis. After that, a soft-thresholding parameter *β* was employed to ensure a scale-free co-expression network. Additionally, hierarchical clustering of the weighting coefficient matrix was also used in WGCNA to identify gene modules, which consisted of a cluster of densely interconnected genes. In order to identify functional modules, the adjacency matrix was transformed into a topological overlap measure (TOM) matrix to estimate its connectivity property in the network (Botía et al., 2017). Average linkage hierarchical clustering was used to construct a clustering dendrogram of the TOM matrix. The minimal gene module size was set to 30 to obtain appropriate modules, and the “DynamicTreeCut” method was used to classify genes with similar expression profiles into the same gene modules (Liao et al., 2021).

Module eigengenes (MEs) and gene significance (GS) were employed to recognize modules related to clinical traits (i.e., tumor stage and histological grade; Chen and Ma, 2021). MEs were considered as the major element of each gene module, and ME expression was recognized on behalf of all genes in a specific module. Thus, the correlation between MEs and clinical traits was analyzed to identify the clinically significant module. Additionally, in the linear regression analysis of clinical characteristics and gene expression profiles, GS was interpreted as the mediating p-value of each gene. Module significance (MS) referred to the average GS across all genes in the module. With the mostly absolute MS, the brown module was defined as the clinically significant module. The functional enrichment analysis of all genes in the brown module was conducted using the “enrichplot” and “ggplot2” R packages.

### Identification of Real Hub Genes Among Patients With Pancreatic Adenocarcinoma

#### Real Hub Gene Identification

After selecting modules of interest, we analyzed GS and module membership (MM, correlation between the module’s own genes and gene expression profiles) for each gene and set their thresholds. The thresholds for screening qualified genes in the module were identified as MM>0.8 and GS>0.2. The qualified genes were selected for uploading into the STRING database for protein–protein interaction (PPI) network construction; meanwhile, the Cytoscape software (version 3.8.2) was utilized for the visualization of the PPI network (Su et al., 2014). Genes with interaction scores >0.4 and node connectivity >3 in the PPI network were identified as candidate genes (Wang et al., 2019; Li et al., 2020a; Zhao et al., 2020). The candidate genes significantly associated with clinical features [i.e., stage, overall survival (OS), and disease-free survival (DFS)] were defined as real hub genes. We uploaded the candidate genes acquired from the PPI network into the gene expression profile interactive analysis database (GEPIA; Tang et al., 2017) to recognize the genes associated with OS, DFS, and stage (i.e., the real hub genes) for further analysis.

### Expression Profile and Independent Prognostic Analyses of Real Hub Genes in Pancreatic Adenocarcinoma

We downloaded the raw data from The Cancer Genome Atlas (TCGA) and the Genotype-Tissue Expression project (GTEx) to perform differential expression analysis of real hub genes between PAAD and normal pancreatic tissues. Among them, 178 PAAD samples obtained from the TCGA database were considered as the experimental group. Four normal pancreatic samples obtained from the TCGA database and 168 normal pancreatic samples obtained from the GTEx database were combined and defined as the control group. All of the data from TCGA and GTEx were converted into the log_2_(x+1) form (Yuan et al., 2021a). The “sva” R package was employed to batch normalize the raw data from different databases. The “stat_compare_means” function in R was performed to explore the differential expressions of real hub genes in PAAD and normal pancreatic samples.

ONCOMINE^2^ database, a translational bioinformatics web server, provides powerful analysis of genomics data (Rhodes et al., 2004). In this study, we set 1.5-fold change as the significance threshold and investigate the differential expression of real hub genes acquired from the TCGA and GTEx databases by Student’s *t* test. The Human Protein Atlas (HPA) database (Uhlen et al., 2015, 2017; Thul et al., 2017) was employed to analyze the discrepancy in the translational level of real hub genes between PAAD and normal pancreatic tissues, and to demonstrate the cellular location of real hub genes in the pancreatic tissues. Moreover, univariate and multivariate Cox regression analyses integrating real hub gene expression with clinicopathological parameters were performed.

#### The Immunoregulatory Effects of Real Hub Genes on the Tumor Microenvironment of Pancreatic Adenocarcinoma

The TIMER^3^ database is a user-friendly and reliable service that can systematically evaluate immune cell infiltration and their clinical effects (Li et al., 2017). It was used to explore the association of real hub genes and the tumor immune microenvironment. “Gene module” was utilized to estimate the relationship of the real hub genes with the six common immune cell infiltrations. The “SCNA module” was utilized to uncover the potential association between somatic copy number alteration (SCNA) of the real hub genes and the immune cell infiltration abundance in PAAD. A correlation coefficient greater than 0.3 obtained from TIMER database often indicates a good correlation.

### Identification and Verification of a Novel Weighted Gene Co-expression Network Analysis-Derived Prognostic Signature

#### Construction and Validation of a Novel Prognostic Panel

In addition to excavating real hub genes in PAAD, we also developed and validated a novel prognostic signature using 49 genes in the brown module. We downloaded data from the International Cancer Genome Consortium (ICGC) databases. Then, the TCGA cohort was considered as the training dataset, whereas the ICGC cohort was identified as the validation cohort. After batch correction *via* the “sva” R package, the expression profiles of 49 genes in both TCGA and ICGC cohorts were obtained for further development of a novel prognostic panel using LASSO-Cox regression analysis. First, univariate Cox regression analysis was utilized to evaluate the prognostic performances of 49 genes in PAAD. Subsequently, we utilized LASSO regression analysis to eliminate colinearity and over-fitting of the genes with the prognostic values. Ultimately, multivariate Cox proportional hazards regression analysis was implemented to construct a novel prognostic signature. Each sample was allocated into high-risk or low-risk subgroups based on the median risk score of the prognostic signature obtained above (risk score=$\sum_{k=1}^{n}expk*\beta k$). For both the training and validation datasets, principal component analysis (PCA) was utilized to show the distribution of each sample in the high- and low-risk subgroups. Kaplan–Meier survival analysis was implemented to analyze the prognostic performance of the model. The receiver operating characteristic (ROC) curve was plotted to verify the diagnostic values of predicting 1- and 3-year survival rates. In order to highlight the superiority and accuracy of our model, we also carefully compared our WGCNA-derived prognostic model with the glycolysis prognostic model constructed by Li et al. (2021) and the m6A prognostic model constructed by Xu et al. (2021). The risk scores of each patient were also obtained according to the risk score computational formula provided by Li et al. (2021) and Xu et al. (2021). The “timeROC” R package was further applied to compute the AUC values of each model.

In addition to these, the chi-square test was used to compare the distribution in terms of stage (Stage IA/Stage IB/Stage IIA/Stage IIB/Stage III/Stage IV), radiation therapy (Yes/No), chronic pancreatitis history (Yes/No), diabetes history (Yes/No), alcohol history (Yes/No), residual tumor (R0/R1/R2/RX), maximum tumor dimension, histologic grade (G1/G2/G3/G4/GX), surgery type (distal pancreatectomy/other method/total pancreatectomy/Whipple), age, sex (Male/Female), neoplasm location (body of the pancreas/head of the pancreas/other/tail of the pancreas), histological type [pancreas–adenocarcinoma–other subtype/pancreas–adenocarcinoma ductal type/pancreas–colloid (mucinous non-cystic) carcinoma/pancreas–undifferentiated carcinoma], and survival state (dead/alive) between the high- and low-risk subgroups.

#### Independent Prognostic Analysis of Risk Score and Establishment of the Nomogram Plot and Calibration Curve

Cox regression analysis was applied to estimate the prognostic factors related to OS in the univariate and multivariate models. The factors significantly related to the prognosis of patients with PAAD in both univariate and multivariate analyses (*p*<0.05) and the well-established prognosis-related factors (i.e., stage and grade) were selected to construct a nomogram plot using the “rms” R package, as well as estimate survival rates. We also established calibration plots to examine the degree of fit between the nomogram-estimated and actual survival probabilities. Through multivariate ROC curves, the prognostic performance of the risk score-based nomogram was also compared with some common clinical parameters, including sex, age, grade, and stage.

#### Exploration of Cellular Processes, Signaling Pathways, Immune Status, and Mutation Profile Influenced by the Weighted Gene Co-expression Network Analysis-Derived Multigene Prognostic Signature

To explore the mechanisms of different prognoses, the “limma” R package was implemented to recognize the differently expressed genes (DEGs, |log_2_FC|>1 and FDR<0.05) between the high- and low-risk subgroups. Gene ontology (GO) enrichment analysis of DEGs was performed using the “clusterProfiler” package. To determine the discrepancy in the activated signaling pathways between the high- and low-risk subgroups, GSEA was performed for the Kyoto Encyclopedia of Genes and Genomes (KEGG) analysis using the GSEA software version 4.0.3. Additionally, the “gsva” R package was used to perform ssGSEA for the identification of the discrepancy in immunocyte infiltration and immune-related functions between the high- and low-risk subgroups. We also investigated the differential expression of certain immune checkpoints in the high- and low-risk subgroups and analyzed the correlation between the expression of immune checkpoints and the risk score computed by our WGCNA-derived multigene prognostic signature. Finally, we obtained the somatic mutation profiles of all PAAD samples from the “Masked Somatic Mutation” category in the TCGA database, which included four types of mutation data based on diverse processing software. We also selected the “Varscan Variant” process for further mutation analysis. Subsequently, we counted the mutations of each gene in each PAAD sample in the high- and low-risk subgroups. Then, the “maftools” R package was used to perform various mutation analyses and provide visualization of the mutation analysis results.

## Results

### Detection Modules Related to Clinical Traits

**Figure 1:**
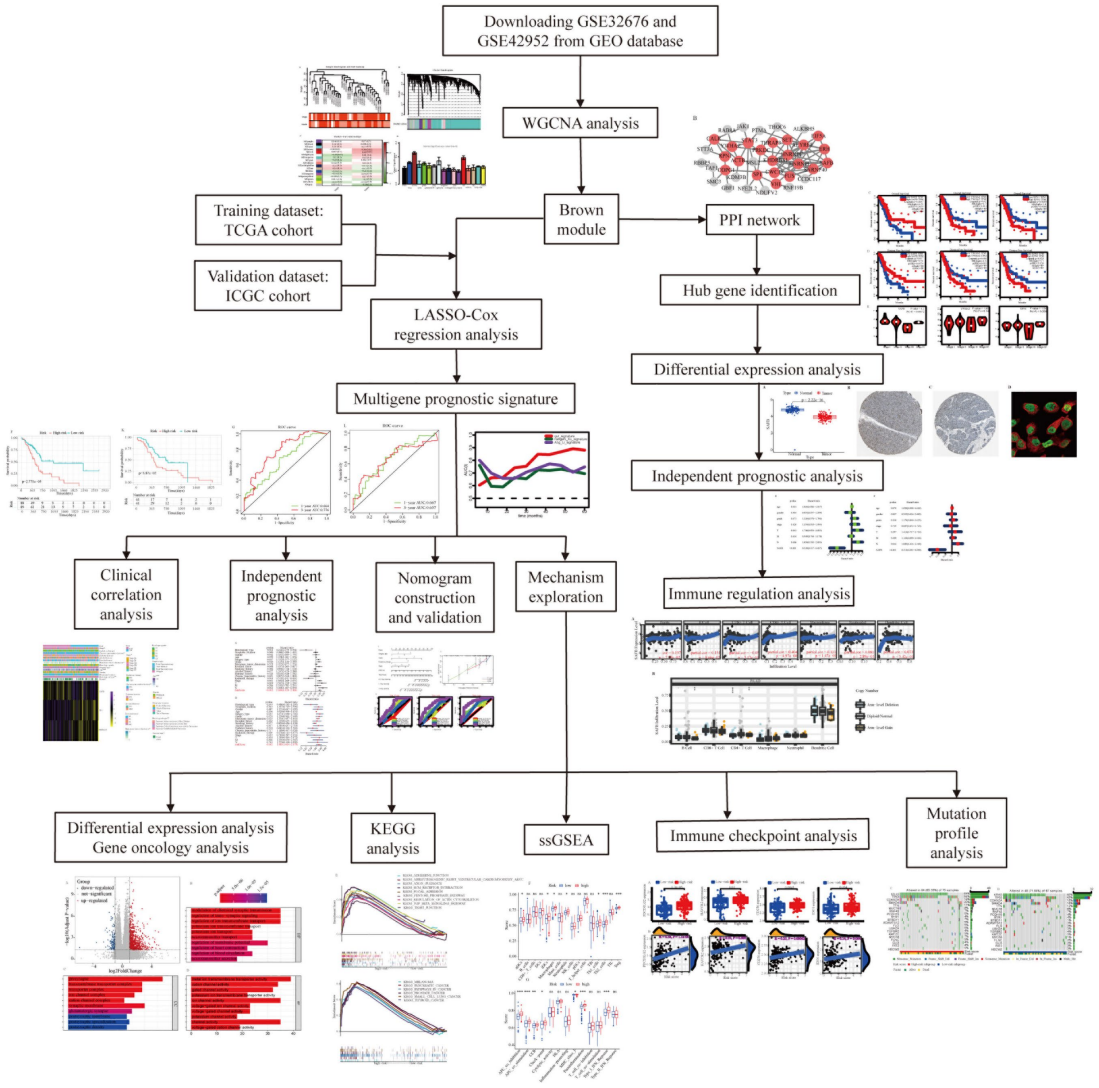
Flow chart of the present study.

**Figure 2:**
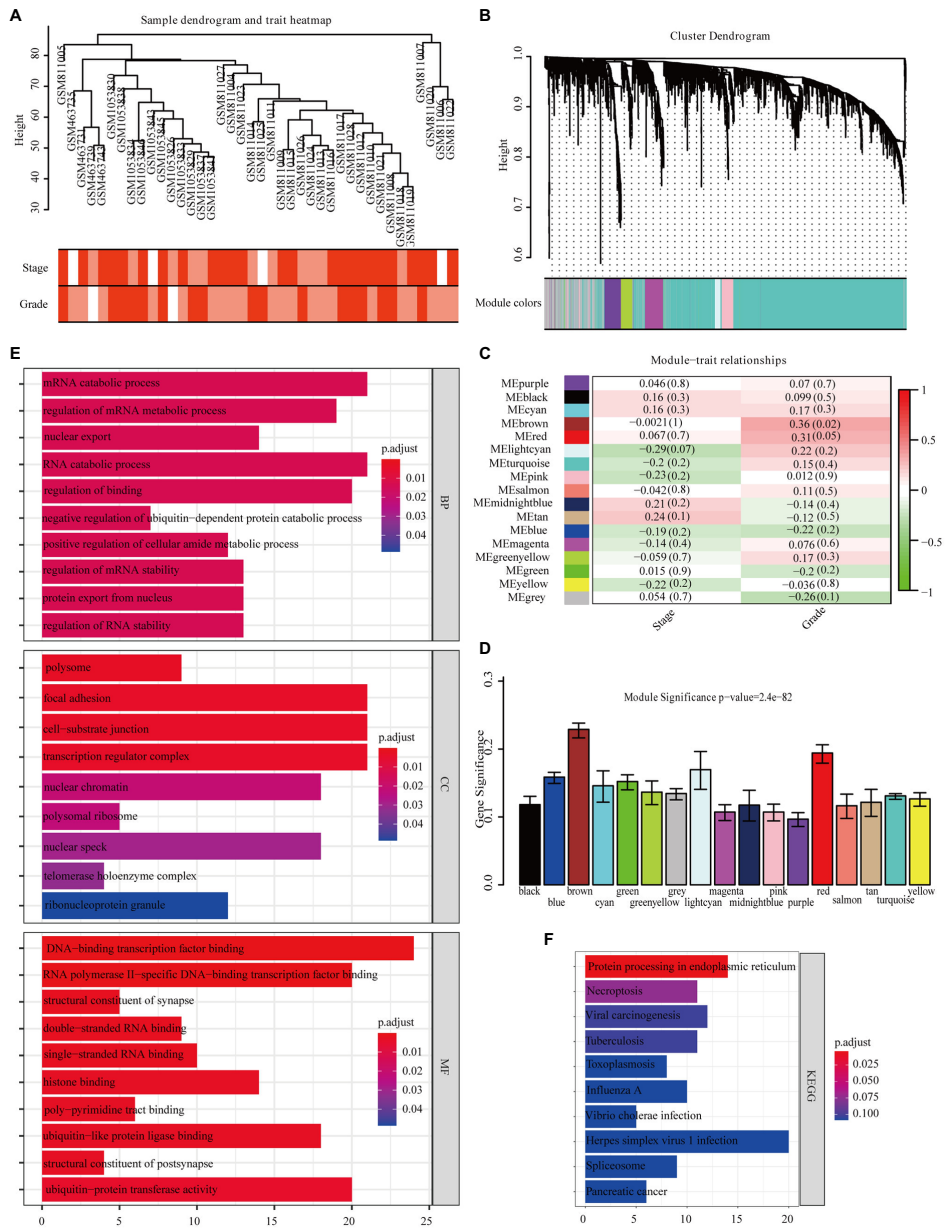
Identification of modules closely related to the clinical characteristics of pancreatic adenocarcinoma (PAAD). **(A)** Clustering dendrogram of 40 samples. **(B)** Dendrogram of all differentially expressed genes clustered based on a dissimilarity measure (1-TOM). **(C)** Correlation heatmap between module eigengenes (MEs) and the clinical characteristics of PAAD. **(D)** Distribution of average gene significance (GS) in 17 modules significantly related to PAAD grade. Functional enrichment analysis of all genes in the brown module [**(E)** Gene ontology, **(F)** Kyoto Encyclopedia of Genes and Genomes (KEGG)].

The overall design of the study is indexed in Figure 1. The GSE32676 and GSE42952 samples were clustered, and there were no outlier samples (Figure 2A). In this research, we set the soft threshold as 7 (scale free *R*^2^=0.88) to make sure that the network was scale free (Appendix A). Through the average linkage hierarchical clustering, we recognized 17 modules in total (Figure 2B). The brown module was most closely related to the tumor grade and was selected as the critical module (Figures 2C,D). Subsequently, functional enrichment analysis of the genes in the brown module revealed that these genes were involved in the mRNA catabolic process, the regulation of the mRNA metabolic process, nuclear export, the negative regulation of the ubiquitin-dependent protein catabolic process, and the regulation of RNA stability in terms of biological process (BP) categories (Figure 2E). These genes were also significantly related to many other processes, including the cellular component (CC) categories of polysome, focal adhesion, cell–substrate junction, transcription regulator complex, and nuclear chromatin (Figure 2E). Additionally, the molecular function (MF) results showed that these genes were mainly involved in DNA-binding transcription factor binding, RNA polymerase II-specific DNA-binding transcription factor binding, double- and single-stranded RNA binding, and histone binding (Figure 2E). Based on the KEGG pathway analysis, our results indicate that these genes were primarily involved in protein processing in the endoplasmic reticulum, necroptosis, and pancreatic cancer (Figure 2F).

### Identification of Real Hub Genes Among Patients With Pancreatic Adenocarcinoma

#### Real Hub Gene Identification

**Figure 3:**
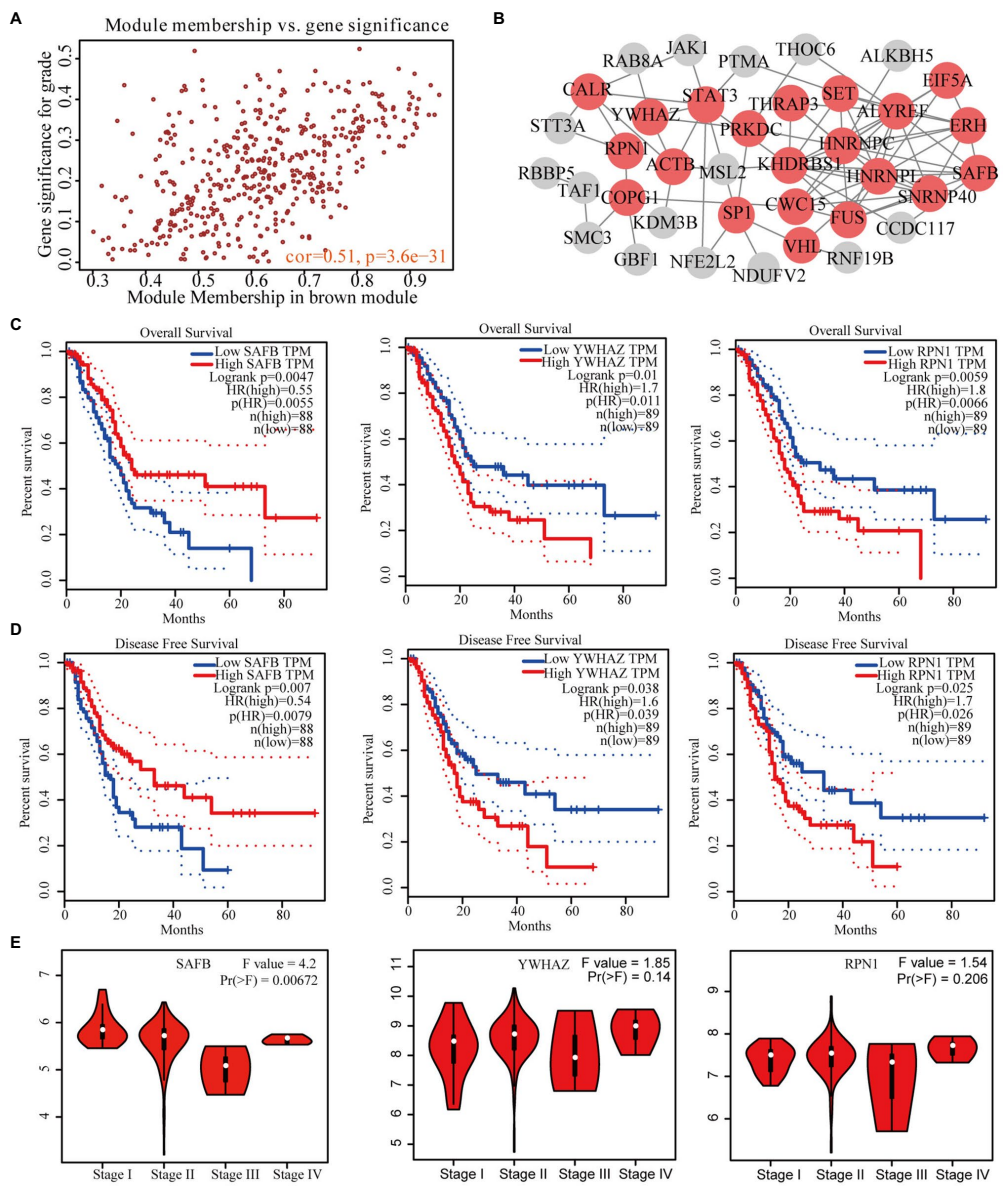
Identification of real hub genes significantly associated with PAAD prognosis and progression. **(A)** Scatter plot of MEs in the brown module. **(B)** Protein–protein interaction (PPI) network of weighted gene co-expression network analysis (WGCNA)-derived hub genes (the red nodes represent the candidate genes in the PPI network). **(C)** Overall survival (OS) and **(D)** disease-free survival (DFS) curves of candidate genes in PAAD gene expression profile interactive analysis (GEPIA). **(E)** Correlation between prognosis-related candidate genes and PAAD stage.

We identified 49 high-connection genes in the brown module based on the predefined criteria (i.e., MM>0.8 and GS>0.2; Figure 3A). Then, we conducted a PPI network analysis by uploading these 49 genes to the STRING database (Figure 3B). Subsequently, 21 candidate genes in the PPI network were preserved based on the predefined criteria (i.e., interaction scores >0.4 and node connectivity >3). GEPIA revealed that three of the 21 candidate genes (i.e., SAFB, YWHAZ, and RPN1) were significantly associated with the OS and DFS of patients with PAAD (Figures 3C,D). However, only SAFB was significantly associated with PAAD stage and was therefore considered as the real hub gene of PAAD (Figure 3E).

#### Expression Profile and Independent Prognostic Analyses of Real Hub Genes in Pancreatic Adenocarcinoma

**Figure 4:**
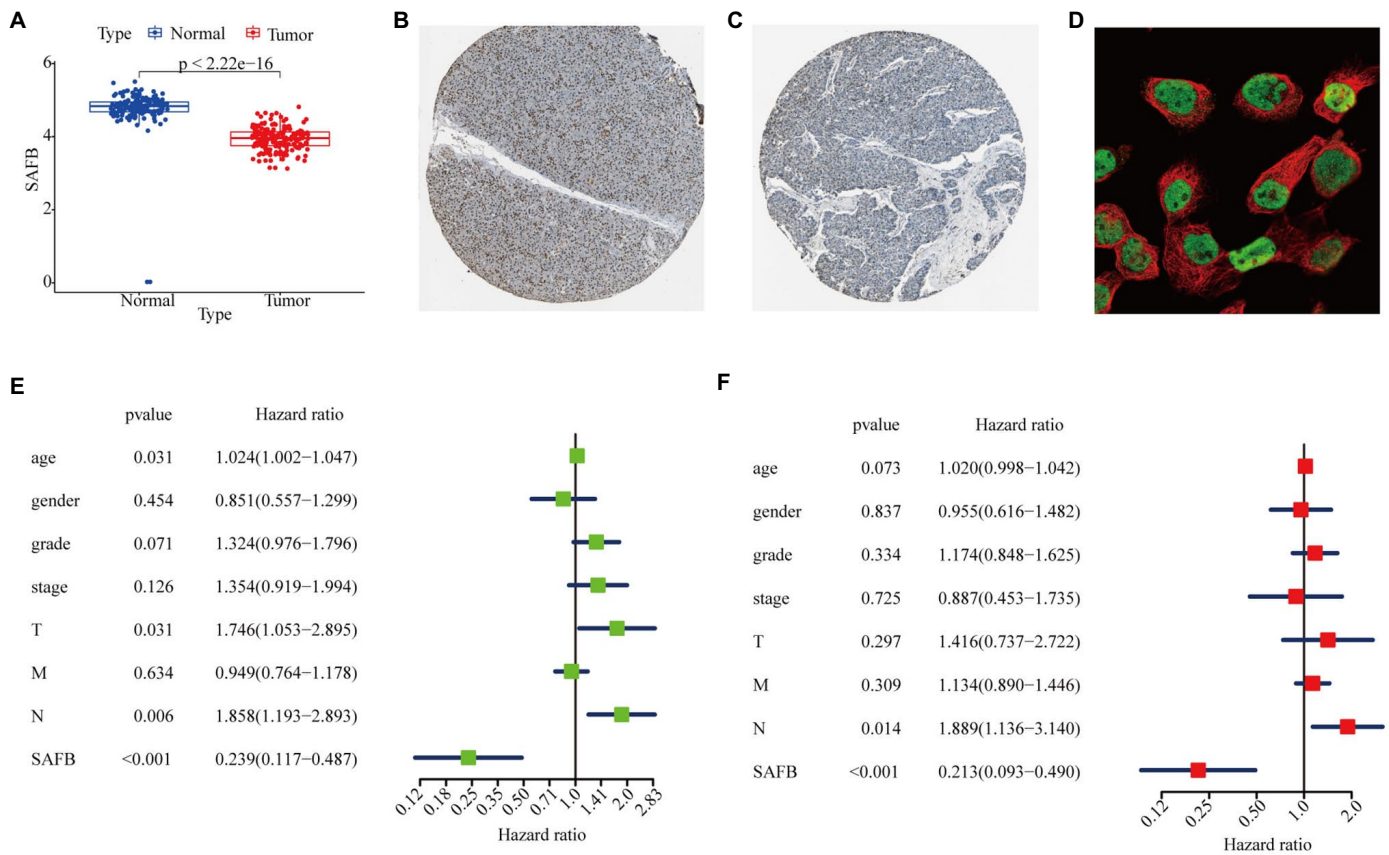
Expression profile and independent prognostic analyses of scaffold attachment factor B (SAFB) in PAAD. **(A)** Differential expression analysis of SAFB between PAAD and normal pancreatic tissues based on The Cancer Genome Atlas (TCGA) and Genotype-Tissue Expression (GTEx) databases. Immunohistochemistry analysis of SAFB based on the Human Protein Atlas (HPA) database. **(B)** Protein levels of SAFB in the normal pancreatic tissue (staining: high; intensity: strong; quantity: >75%). **(C)** Protein levels of SAFB in the PAAD tissue (staining: low; intensity: moderate; quantity: <25%). **(D)** Cellular location of SAFB expression. **(E)** Univariate Cox regression analysis of the association among SAFB expression, clinicopathological parameters, and the OS of patients in the TCGA cohort. **(F)** Multivariate Cox regression analysis of the association among SAFB expression, clinicopathological parameters, and the OS of patients in the TCGA cohort.

We intensively studied the expression pattern of SAFB between tumor and non-tumor tissues based on the TCGA and GTEx databases. Compared with non-tumor tissues, SAFB mRNA was significantly lower in PAAD (Figure 4A). Subsequently, the ONCOMINE database was used to verify the results, which led to a similar conclusion (Appendix B). Moreover, compared with normal pancreatic tissues, the translational levels of SAFB were also significantly lower in PAAD tissues based on the HPA database (Figures 4B,C). The expression product of SAFB was mainly located on the nucleoplasm (Figure 4D).

In order to confirm whether SAFB acted to predict prognosis independently, univariate and multivariate Cox regression analyses were performed. The univariate Cox analysis showed that SAFB was significantly correlated with OS (HR=0.239, 95% CI=0.117–0.487, *p*<0.001; Figure 4E). After correcting for other confounding factors including age, sex, grade, and stage, the multivariate Cox analysis showed that SAFB was still an independent predictor for OS (HR=0.213, 95% CI=0.093–0.490, *p*<0.001; Figure 4F). As outlined above, SAFB could serve as an independent prognostic indicator and aid in the survival of patients with PAAD.

#### The Immunoregulatory Effects of Real Hub Genes on the Tumor Microenvironment of Pancreatic Adenocarcinoma

**Figure 5:**
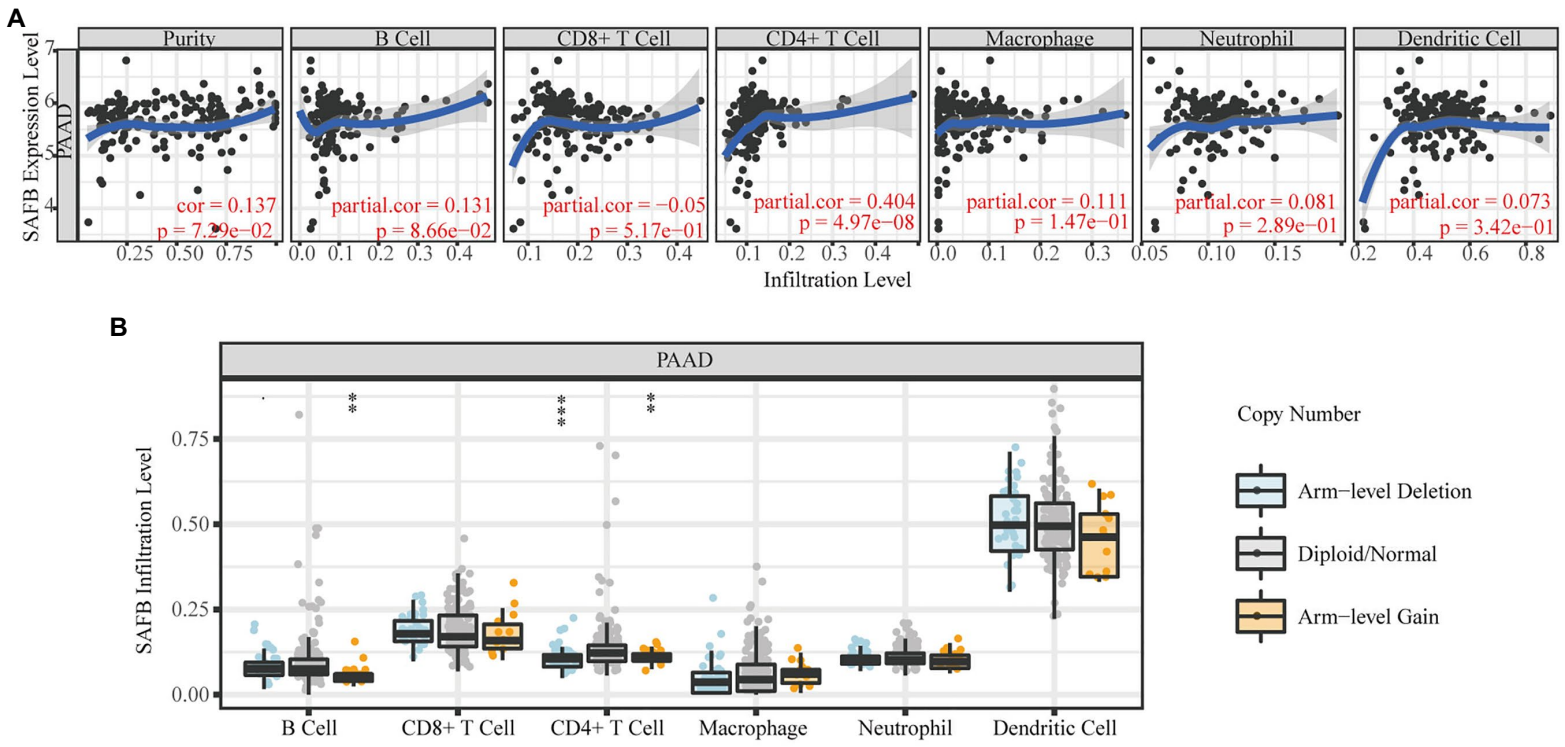
The immunoregulatory effects of SAFB on the PAAD tumor microenvironment. **(A)** The relationships between SAFB expression and the abundance of six common immunocyte infiltrations in PAAD. **(B)** The relationships between somatic copy number alteration of SAFB and the abundance of six common immunocyte infiltrations in PAAD. ^*^p < 0.05; ^**^p < 0.01; and ^***^p < 0.001.

The TIMER database was utilized to explore the relationship between SAFB expression and immune cell infiltration. SAFB expression moderately correlated with CD4^+^ T cell infiltration (Cor=0.404, *p*=4.97^−08^; Figure 5A). Moreover, different SCNAs of SAFB were closely related to CD4^+^ T cells and B cells. We speculate that SAFB was involved with the PAAD immune microenvironment, thus, influencing the clinical outcomes of patients with PAAD (Figure 5B).

### Identification and Verification of a Novel Weighted Gene Co-expression Network Analysis-Derived Prognostic Signature

#### Prognostic Panel Construction and Evaluation

**Figure 6:**
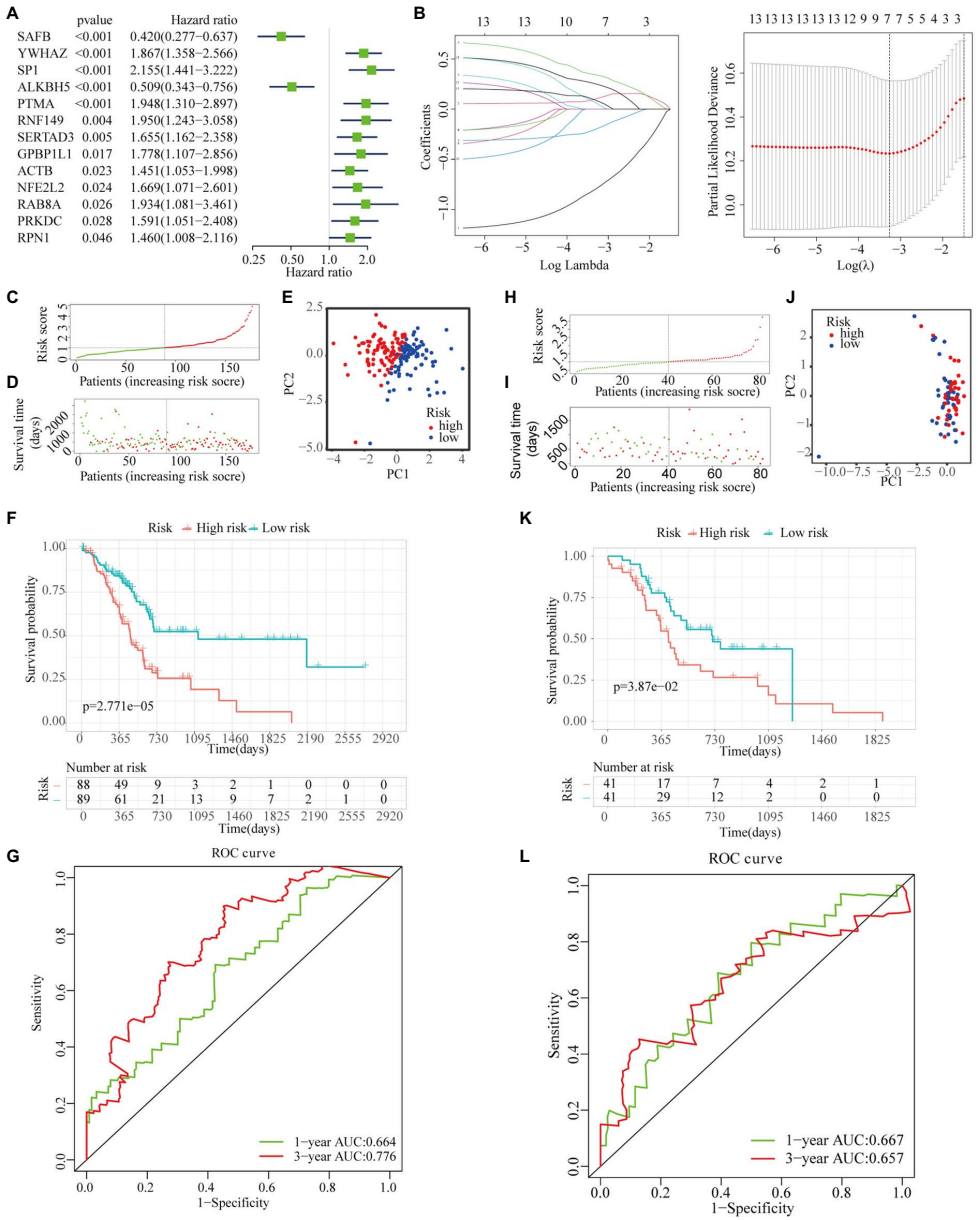
Construction and validation of a novel multigene prognostic signature. **(A)** Univariate Cox regression analysis of all the genes in the brown module. **(B)** The coefficients calculated by the LASSO regression analysis are shown in the TCGA cohort. **(C)** The distribution and median value of the risk scores in the TCGA cohort. **(D)** The distributions of OS status, OS, and risk score in the TCGA cohort. **(E)** The principal component analysis (PCA) plot of TCGA cohort. **(F)** The Kaplan–Meier curves for the OS of patients in the high- and low-risk subgroups in the TCGA cohort. **(G)** The AUC of time-dependent receiver operating characteristic (ROC) curves in the TCGA cohort. **(H)** The distribution and median value of the risk scores in the International Cancer Genome Consortium (ICGC) cohort. **(I)** The distribution of OS status, OS, and risk score in the ICGC cohort. **(J)** The PCA plot of the TCGA cohort. **(K)** The Kaplan–Meier curves for the OS of patients in the high- and low-risk groups in the ICGC cohort. **(L)** The AUC of the time-dependent ROC curves in the ICGC cohort.

Univariate Cox regression analysis was performed to investigate the prognostic performances of 49 high-connective genes in the brown module, and a total of 13 genes (hazard ratio >1) were found to be significantly related to PAAD prognosis (Figure 6A). The LASSO regression analysis of these 13 genes was performed to avoid over-fitting of the model (Figure 6B). Subsequently, a total of seven grade-related genes (i.e., SAFB, YWHAZ, SP1, ALKBH5, SERTAD3, RAB8A, and RPN1) were preserved for further multivariate Cox regression analysis. Ultimately, a novel multigene PAAD prognostic signature was constructed by integrating three grade-related gene expressions (i.e., SAFB, SP1, and SERTAD3). Based on the Cox coefficient of these three grade-related genes, the prognostic risk score was calculated for each patient in the TCGA cohort as follows: (0.56708507682292×SP1 expression)+(0.448867415920488×SERTAD3 expression)−(1.14695469587815×SAFB expression). All patients were then stratified into high- and low-risk subgroups based on their median risk score (Figure 6C). An increasing number of deaths was observed with the increase in risk score (Figure 6D). The PCA results showed that our prognostic signature could precisely distinguish patients with PAAD into the high- and low-risk subgroups (Figure 6E). A Kaplan–Meier curve was also plotted for the TCGA cohort according to the median risk score, with the high-risk subgroup showing poorer OS than the low-risk subgroup (Figure 6F). Time-dependent ROC curves revealed that the prognostic signature had superior predictive accuracy in the TCGA cohort, with the AUC values for 1- and 3-year survival being 0.664 and 0.776, respectively (Figure 6G). For patients with PAAD whose survival time was >20months, our WGCNA-derived prognostic signature showed a higher predictive accuracy compared with the well-established prognostic signatures constructed by Li et al. and Xu et al. (Li et al., 2021; Xu et al., 2021; Appendix C).

**Figure 7:**
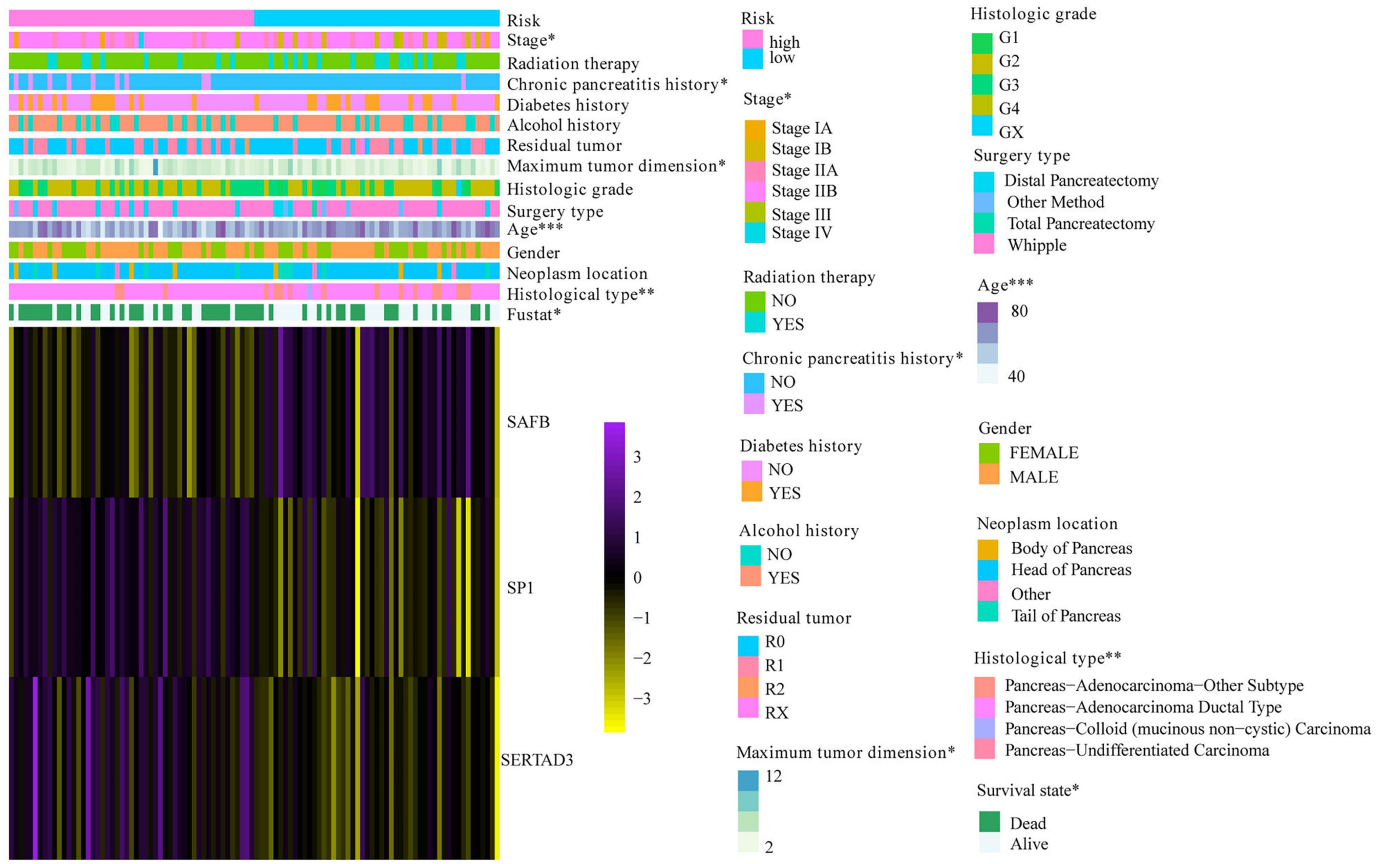
The correlation between risk score and PAAD clinical characteristics. ^*^p < 0.05; ^**^p < 0.01; and ^***^p < 0.001.

To verify the reliability of the WGCNA-derived multigene prognostic signature, we also performed an external verification using the ICGC cohort (Figure 6H). Likewise, the survival rate of patients with PAAD decreased with increasing risk score (Figure 6I). Although the PCA results failed to clearly distinguish patients with PAAD into the high- and low-risk subgroups (Figure 6J), the survival curve showed that there was still a significant statistical difference in prognosis between the high- and low-risk subgroups (Figure 6K). As shown in Figure 6L, the AUC of the prognostic signature for 1- and 3-year survival was 0.667 and 0.657, respectively. In addition, the prognostic signature that we constructed was not only related to the survival state of PAAD, but was also significantly associated with age, stage, chronic pancreatitis history, maximum tumor dimension, and histological type (Figure 7).

#### Independent Prognostic Analysis of Risk Score and Establishment of the Nomogram Plot and Calibration Curve Based on The Cancer Genome Atlas Cohort

**Figure 8:**
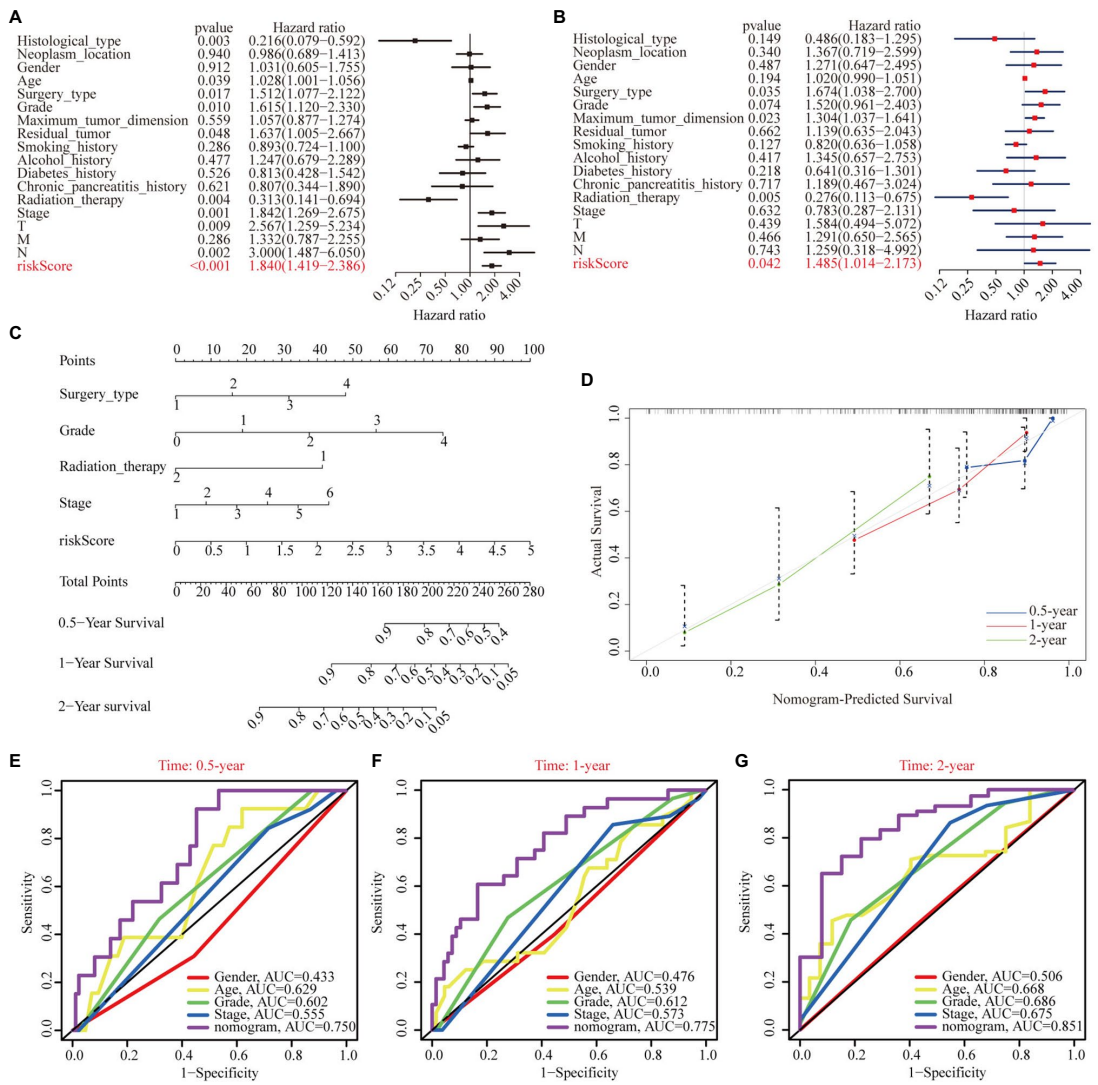
Independent prognostic analysis of risk scores. **(A)** Univariate Cox regression analyses of the association among risk score, clinicopathological parameters, and OS of patients in the TCGA cohort. **(B)** Multivariate Cox regression analyses of the association among risk score, clinicopathological parameters, and OS of patients in the TCGA cohort. **(C)** The nomogram composed of surgery type, histological grade, radiation therapy, stage, and risk score for the prediction of 0.5-, 1-, and 2-year OS probability. **(D)** The calibration plot for the evaluation of the nomogram in predicting 0.5-, 1-, and 2-year OS probability. The multi-index ROC curves integrating nomogram, age, sex, grade, and stage for the prediction of 0.5-year **(E)**, 1-year **(F)**, and 2-year **(G)** OS probability.

To justify whether the prognostic signature could serve as an independent prognostic indicator, univariate and multivariate Cox regression analyses were performed based on the TCGA cohort. The results revealed that the risk score independently predicted the clinical outcomes of patients with PAAD (univariate HR=1.84, 95% CI=1.419–2.386, *p*<0.001; multivariate HR=1.485, 95% CI=1.014–2.173, *p*=0.042; Figures 8A,B). In addition, we found that surgery type and radiation therapy were also independent prognostic factors. Subsequently, a prognostic nomogram plot comprising these independent prognostic indicators (i.e., risk score, surgery type, and radiation therapy) and the well-established prognostic factors (i.e., stage and grade) was constructed as a quantitative tool for predicting OS in patients with PAAD (Figure 8C). The calibration curve indicated that the nomogram performed with great accuracy (Figure 8D). The results of the multivariate ROC analysis also showed that the nomograph based on the risk score derived from WGCNA and LASSO algorithms was significantly better than many clinical parameters, including the AJCC stage and histological grade, in predicting the prognosis of patients with PAAD. The AUC value of the nomogram ROC curve was 0.750, 0.775, and 0.851 for the 0.5-, 1-, and 2-year survival probability of patients with PAAD, respectively (Figures 8E–G).

#### Exploration of Cellular Processes, Signaling Pathways, Immune Status, and Mutation Profile Influenced by the Weighted Gene Co-expression Network Analysis-Derived Multigene Prognostic Signature

**Figure 9:**
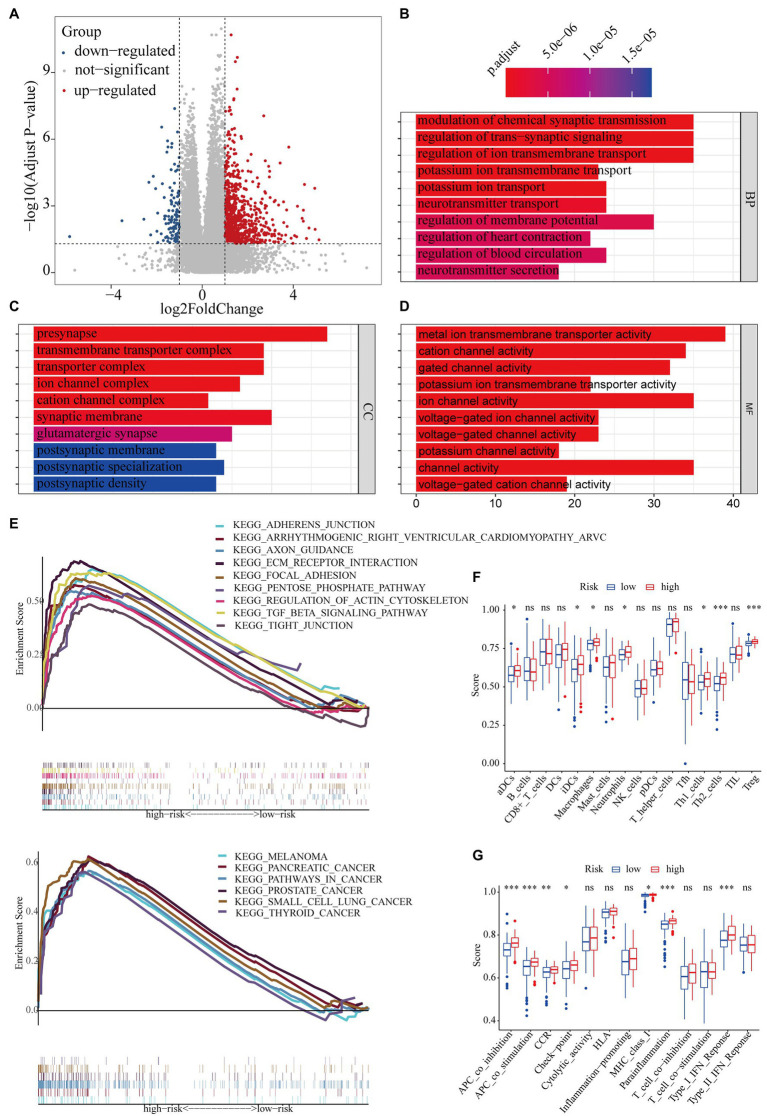
The potential mechanisms of different clinical outcomes between the high- and low-risk subgroups. **(A)** The volcano plot of differentially expressed genes between the high- and low-risk groups. Gene ontology (GO) enrichment analysis for differentially expressed genes between the high- and low-risk groups. **(B)** Biological process (BP), **(C)** cellular component (CC), **(D)**: molecular function (MF). **(E)** Gene set enrichment analysis (GSEA): 15 pathways were identified to be significantly activated in the high-risk group. Comparison with the single-sample gene set enrichment analysis (ssGSEA) scores between the different risk groups. The scores of **(F)** 16 immune cells and **(G)** 13 immune-related functions are displayed in boxplots. ^*^p < 0.05; ^**^p < 0.01; and ^***^p < 0.001.

A total of 1,215 DEGs (358 upregulated and 857 downregulated genes) were recognized between the high- and low-risk subgroups (Figure 9A). Subsequently, functional enrichment analysis was applied to annotate DEG functions, and several cancer-related biological processes were identified, including modulation of the chemical synaptic transmission, regulation of the trans-synaptic signaling, and regulation of the ion transmembrane transport (Figure 9B). In addition, the CC results indicate that DEGs were mainly involved in the transmembrane transporter complex and the ion channel complex (Figure 9C). Similarly, the MF results showed that DEGs played a pivotal role in metal ion transmembrane transporter activity, cation channel activity, and gated channel activity (Figure 9D). GSEA analysis indicated that the adherence junction, the ECM receptor interaction, the transforming growth factor β (TGF-β) signaling pathway, the pentose phosphate pathway, pancreatic cancer, and pathways in cancer were mainly enriched in the high-risk subgroup (Figure 9E).

To further investigate the discrepancy in immune status between the high- and low-risk subgroups, we performed ssGSEA analysis. The antigen presentation capacity significantly differed between the high- and low-risk subgroups (Figures 9F,G). Specifically, macrophages, aDCs, and iDCs, as part of the classical antigen-presenting cells (APCs), had higher scores in the high-risk subgroup (Figure 9F). Part of the APC target cell (i.e., CD4^+^ T cells) also showed higher scores in the high-risk subgroup (Figure 9F). Moreover, the scores of APC-related functions was significantly higher in the high-risk subgroup (Figure 9G). In addition, the scores of Treg, cytokine–cytokine receptor interaction, parainflammation, and type I IFN response were also significantly higher in the high-risk subgroups.

**Figure 10:**
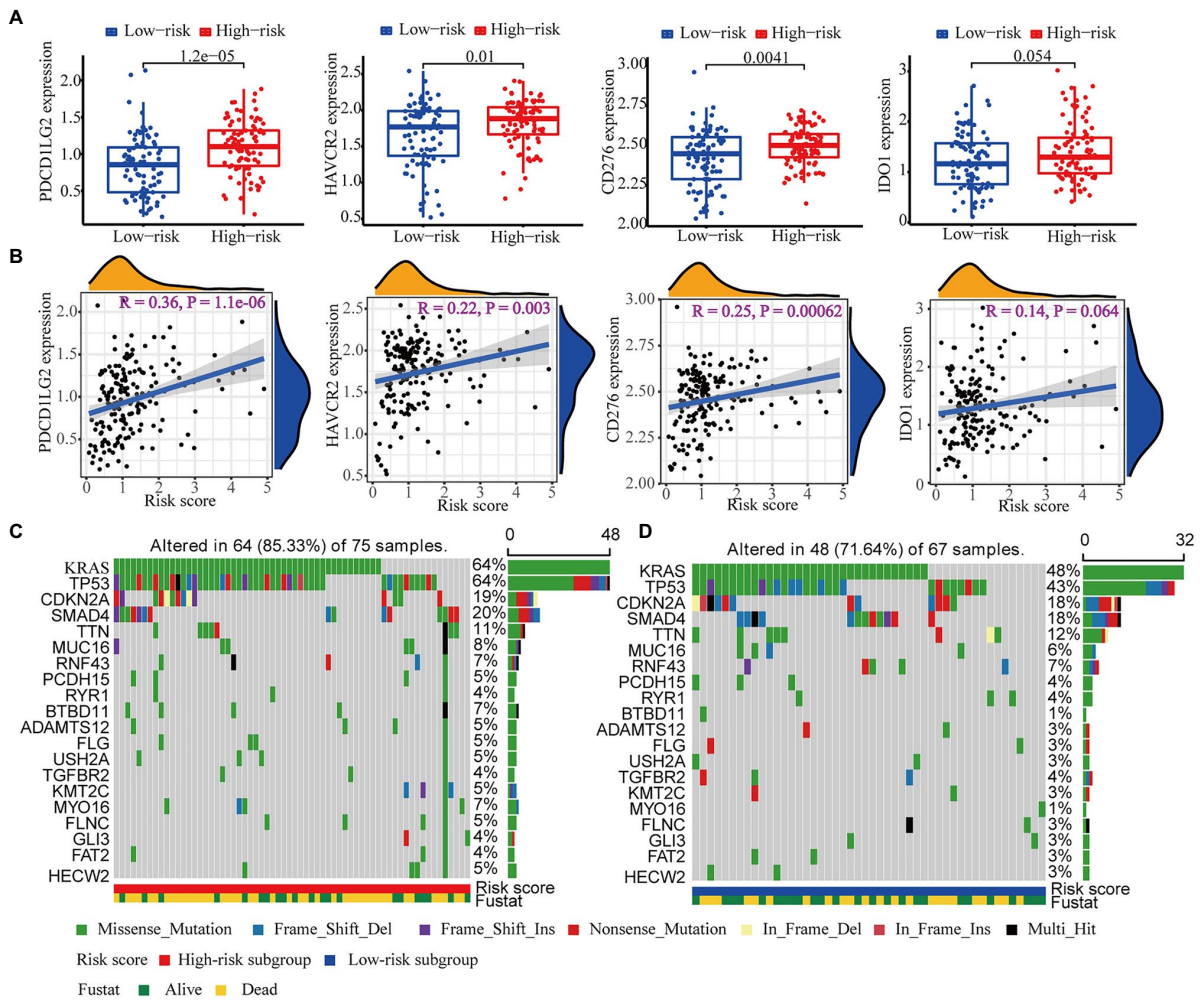
Immune checkpoint and mutation profile analyses. **(A)** The differential expression of PDCD1LG2, HAVCR2, CD276, and IDO1 between the high- and low-risk subgroups. **(B)** The correlation analysis of the expression of PDCD1LG2, HAVCR2, CD276, and IDO1 and the risk score computed by our WGCNA-derived prognostic signature. The top 20 mutational genes in the **(C)** high-risk group and the **(D)** low-risk group are listed.

Since drugs targeting immune checkpoints have been shown to achieve antitumor effects by reversing the immunosuppressive effects of tumors, the expression of immune checkpoints has attracted widespread attention as a biomarker for identifying patients with malignancies to receive immunotherapy (Topalian, 2017). Thus, we subsequently investigated the potential association of immune checkpoints and the risk score computed by our WGCNA-derived multigene prognostic signature. Compared with those in the low-risk subgroup, patients with PAAD in the high-risk subgroup exhibited enhanced expression of PDCD1LG2, HAVCR2, CD276, and IDO1 (Figure 10A). Furthermore, the risk score of patients with PAAD was positively correlated with the expression of these immune checkpoints (Figure 10B). These findings indicate that our WGCNA-derived prognostic signature identification of the high-risk population might benefit from some immune checkpoint inhibitors, to a certain extent.

It has also been reported that mutation profiles had the potential to predict immunotherapy response. Therefore, we further assessed the discrepancy in the mutation profiles between the high- and low-risk subgroups. Figures 10C,D depicts the top 20 mutated genes (e.g., KRAS, TP53, CDKN2A, SMAD4, and TTN) in the high- and low-risk subgroups. Interestingly, the high-risk subgroup showed a higher mutation frequency of genes than the low-risk subgroup, suggesting that the high-risk subgroup might be adapted to immunotherapy.

## Discussion

PAAD is one of the common digestive system malignancies with a poor prognosis, and its incidence and mortality are rising worldwide. The rapid development of bioinformatics provides new insights for exploring the pathogenic mechanism of PAAD. Emerging genes and signaling pathways are considered as the key regulators of PAAD tumorigenesis (Jie et al., 2021; Zheng et al., 2021). An increasing number of diagnostic and prognostic modules have also been identified and verified for PAAD (Li et al., 2020b; Yue et al., 2020). However, the pathophysiological mechanisms of PAAD remain unclear, and the prognostic evaluation of patients with PAAD remains poor. Therefore, we sought to explore the real hub genes to better understand the pathophysiology of PAAD and construct a specific multigene prognostic signature to better evaluate PAAD clinical outcomes.

In this study, we identified the brown module most related to PAAD histological grade using WGCNA. Then, a total of 21 candidate genes were maintained after establishing the PPI network. Among them, we revealed that SAFB was involved in PAAD prognosis and progression, and was considered as the real hub gene of PAAD.

The full name of SAFB is scaffold attachment factor B, which is highly conserved in evolution (Luo et al., 2021). It was first identified through its ability to bind to DNA elements in the scaffold attachment region; however, more attention was subsequently given to RNA binding and protein–protein interaction (Norman et al., 2016; Huo et al., 2020; Zhou et al., 2020). Currently, SAFB is considered as a member of the RNA-binding protein family (Li et al., 2019b). According to reports, SAFB is involved in a variety of biologic behaviors, including DNA repair, apoptosis, stress response, processing of mRNA and regulatory RNA, and interaction with chromatin modification complexes (Oesterreich, 2003; Hong et al., 2012). SAFB expression is high in normal colorectal tissues and is low in colorectal cancer, and this low expression of SAFB predicted a worse outcome for patients with colorectal cancer (Jiao et al., 2017). In addition, low SAFB expression was also predictive of poorer prognosis in patients with breast cancer without adjuvant therapy (Hammerich-Hille et al., 2010). Likewise, our results showed that both transcriptional and translational levels of SAFB were significantly lower in PAAD tissues than in normal pancreatic tissues. Low SAFB expression was also closely associated with the poor prognoses of patients with PAAD. Overall, these data suggest that SAFB might serve as a protective role for various cancers, including colorectal cancer, breast cancer, and PAAD.

Emerging evidence indicates that immune cell infiltration has the potential to influence tumor occurrence and development, and serve as a determining factor of both immunotherapy response and prognoses (Bindea et al., 2013; Liu et al., 2017). In our research, SAFB expression moderately correlated with CD4^+^ T-cell infiltration. Moreover, different SCNAs of SAFB could influence the infiltration of CD4^+^ T cells and B cells. These findings reveal that SAFB might also reflect tumor immunity status. As outlined above, SAFB might improve clinical outcomes *via* the migration of CD4^+^ T cells into the pancreatic tumor microenvironment.

Furthermore, 49 genes in the brown module have been selected to construct a multigene prognostic signature. After performing LASSO-Cox regression analysis, we identified and verified a novel independent prognostic signature comprising SAFB, SP1, and SERTAD3 that were tightly related to PAAD prognoses. SP1, as the forerunner of the SP family, is highly expressed in various tumors and is significantly associated with a poor prognosis (Beishline and Azizkhan-Clifford, 2015). SERTAD3 has the potential to contribute to tumor growth by inducing E2F activity (Darwish et al., 2007). Based on our novel prognostic signature, patients with PAAD could be split into high- and low-risk subgroups with different prognoses. The ROC curves for 1- and 3-year survival rates also proved the predictive accuracy of our prognostic signature. More importantly, our results imply that the predictive accuracy of our signature was superior to the prognostic model constructed by Li et al. (2021) and Xu et al. (2021), especially in terms of predicting the 3- and 5-year survival probabilities of patients with PAAD.

The univariate and multivariate Cox regression analyses also proved the independent prognostic ability of our signature. We established a specific nomogram consisting of risk score, surgery type, grade, radiation therapy, and stage to quantitatively evaluate the survival probability of patients with PAAD. More importantly, the calibration plots showed that the survival probability predicted by our nomogram was in accordance with the actual survival condition. Moreover, the multi-index ROC curves demonstrated that the predictive ability of our nomogram was superior to the well-established AJCC stage and histological grade. These suggest that our nomogram was robust and had tremendous clinical application prospect, and could help clinicians better carry out personalized intervention and change the treatment strategy according to specific conditions. In addition to these, functional enrichment analysis, GSEA, and ssGSEA were performed to uncover the potential mechanisms of different prognoses between the different subgroups. Notably, many tumor-related signal pathways were enriched in high-risk subgroup, which were responsible for the poorer prognosis of the patients belonging to this subgroup.

It has been well established that anti-cancer and cancer-promoting immune response often simultaneously exist in the local tumor tissue. Likewise, our results showed that both anti-tumor immune response and immune escape were amplified in the high-risk subgroup. It is generally accepted that APCs act to enhance immune response through enhancement of antigen recognition and activation of CD4^+^ T cells. Interestingly, the scores of APCs, target cells of APCs, and APC-related functions were observed to be significantly higher in the high-risk subgroup, suggesting the amplification of anti-tumor immune response. Similarly, the Treg score was significantly higher in the high-risk subgroup. Emerging evidence indicate that Treg has the potential to help tumor cells evade immune system surveillance by inhibiting local immune response. Overall, dysregulation of immune response might contribute to poorer prognosis in high-risk patients with PAAD.

The expression of certain immune checkpoints (such as PDCD1LG2, HAVCR2, CD276, and IDO1) also played an important role in the regulation of immune response by inhibiting the activation of anti-tumor immunocytes and inducing immune escape (Okazaki et al., 2013; Tang et al., 2020). Thus, in our study, it is easy to explain why the expression of immune checkpoint molecules is higher in the high-risk subgroup and why the WGCNA-derived risk signature positively correlates with the expression of immune checkpoint molecules. We reason that the poorer prognosis of patients with PAAD in the high-risk subgroup could be attributed to the enhanced expression of immune checkpoints. It has been well established that higher expression of immune checkpoint molecules usually benefits more from immune checkpoint inhibitors (ICIs; Peng et al., 2021). Overall, these data indicate that patients with PAAD in the high-risk subgroup might have improved prognoses through the application of ICIs.

A large number of studies have shown that the burden of tumor mutation is closely related to the efficacy of immunotherapy (Hugo et al., 2016; June et al., 2018). The more mutated genes are present in tumor cells, the more mutation-related mRNAs and proteins may be produced, which can be recognized and targeted by the immune system (Zhang et al., 2021). Therefore, the high mutation frequency of genes is associated with improved immunotherapy responses, long-lasting clinical benefits, and progression-free survival (Rizvi et al., 2015). In 2020, Cai et al. (2020) reported that higher mutation frequency was closely associated with the poorer clinical outcomes of patients with hepatocellular carcinoma. Likewise, our data indicated that patients with PAAD in the high-risk subgroup with high gene mutation frequency had worse prognoses. It is generally accepted that KRAS serves as an important member of the oncogene family and could be activated by EGFR (Petroni et al., 2021). Mutant KRAS can be continuously activated without ligands and then facilitate tumorigenesis through the establishment of an immunosuppressive microenvironment (Spranger and Gajewski, 2018; Petroni et al., 2021). TP53 is a classic tumor suppressor gene (Olivier et al., 2010). However, when the p53 gene is mutated, due to the change in spatial conformation, p53 loses its anti-tumor function and the mutation itself also endows p53 with the function of an oncogene by inhibiting the innate immune signaling (Ghosh et al., 2021). The mutation or deletion of the CDKN2A gene also gives cells the ability for unlimited reproduction (Cicenas et al., 2017). Moreover, SMAD4 serves as the central mediator TGF-β signaling, and its mutation plays an important initiating role by disrupting DNA damage response and repair mechanisms and enhancing genomic instability, suggesting its distinct roles in different tumor types (Zhao et al., 2018). Furthermore, the high expression of the MUC16 C terminal (MUC16c) reflects the high proportion of Treg in the tumor microenvironment, to some extent (Fan et al., 2018). MUC16c participates in the regulation of Treg differentiation by promoting the expression and secretion of IL-6, which promotes the immune escape of pancreatic cancer (Fan et al., 2018). As alluded above, the effect caused by mutations of KRAS, TP53, CDKN2A, SMAD4, and MUC16 were in accordance with risks predicted by our WGCNA-derived prognostic signature. More importantly, the high mutation frequency in the high-risk subgroup could increase the clinical efficacy of immunotherapy.

Nonetheless, our study has the following limitations that should be noted. First, the number of samples we retrieved from the GEO and TCGA databases was relatively small; therefore, our results should be verified in multicenter clinical trials and prospective studies for better clinical application. Second, our current study is a bioinformatics research, and further basic experiments are warranted to validate our results.

In conclusion, through integrally analyzing a multitude of databases, one real hub gene (i.e., SAFB) that could truly serve as a novel progression and prognosis indicator of PAAD was identified in our study. In addition, a novel multigene independent prognostic signature consisting of SAFB, SP1, and SERTAD3 was also successfully constructed and validated to better predict the prognosis of PAAD. Furthermore, a specific nomogram comprising risk score, surgery type, grade, radiation therapy, and stage was developed to precisely predict the survival probability of patients with PAAD. Overall, these findings provide new insights for the exploration of the PAAD pathogenesis, as well as valuable information for clinical decision making and precision medicine.

## Data Availability Statement

The original contributions presented in the study are included in the article/supplementary material; further inquiries can be directed to the corresponding authors.

## Author Contributions

The authors alone are responsible for the content and writing of the paper. QY contributed to the design of the study, collection and interpretation of data, and drafting and revising of the manuscript. JR, ZW, and LJ participated in the design of the study, collection of data, and drafting of the manuscript. DD and DS conceived the study and reviewed and edited the manuscript. All authors contributed to the article and approved the submitted version.

## Conflict of Interest

The authors declare that the research was conducted in the absence of any commercial or financial relationships that could be construed as a potential conflict of interest.

## Publisher’s Note

All claims expressed in this article are solely those of the authors and do not necessarily represent those of their affiliated organizations, or those of the publisher, the editors and the reviewers. Any product that may be evaluated in this article, or claim that may be made by its manufacturer, is not guaranteed or endorsed by the publisher.

## Footnotes

^1^ https://www.ncbi.nlm.nih.gov/geo/

^2^ www.oncomine.org

^3^ https://cistrome.shinyapps.io/timer/

## Supplementary Material

Appendix A Soft-thresholding power screening. **(A)** The scale-free fit index for soft-thresholding powers (β). **(B)** Mean connectivity calculation. **(C)** Checking the scale-free topology when β = 7.

Appendix B The mRNA level of SAFB in tumor and non-tumor tissues (ONCOMINE).

Appendix C The AUC values of the ROC curve for the predictive performance of our signature compared with the prognostic signatures constructed by Li et al. (2021) and Xu et al. (2021).
